# Neurodevelopmental Consequences of Antenatal Exposure to Antipsychotic Medication: A Systematic Review

**DOI:** 10.1192/j.eurpsy.2025.365

**Published:** 2025-08-26

**Authors:** A.-S. Rommel, T. Robakis, C. Kaplan, E. Poels

**Affiliations:** 1 Psychiatry, Icahn School of Medicine, New York, United States; 2 Psychiatry, Erasmus MC, Rotterdam, Netherlands

## Abstract

**Introduction:**

Antipsychotic (AP) medications are increasingly prescribed, with indications ranging from psychotic illness to mood disorders. Many patients and clinicians are concerned about long-term consequences of drug exposure in the neurodevelopmentally critical prenatal window. Yet maternal mental illness itself is also associated with significant changes to the prenatal environment.

**Objectives:**

To systematically review the literature on the short- and long-term behavioral, socioemotional, psychomotor, and neurocognitive outcomes in children prenatally exposed to AP medication.

**Methods:**

We included original studies assessing cognitive, motor, behavioral, social, and psychiatric. Searches were performed in MEDLINE, Cochrane, Embase, and PsycINFO for studies up to March 15, 2024. Quality and risk of bias were assessed using the Newcastle Ottawa Scale (NOS). Studies were eligible for inclusion if they were original, peer-reviewed research which assessed human offspring of any age, prenatally exposed to any antipsychotic medication, regardless of maternal indication for use.

**Results:**

We identified 1,315 studies, and reviewed 53 in full-text screening. The final synthesis included 16 studies (6 cohort and 10 register-based studies) with the number of prenatally exposed individuals ranging from 17 to >15,000.Eleven studies included a control group with maternal mental illness in at least one of their analyses. These groups varied with some including any maternal mental illness, while others used an antipsychotic discontinuation control, and still others used non-antipsychotic psychiatric medication use as a control. Five studies included only a general or healthy maternal population control. Eight studies assessed motor development, ranging from newborn assessments up to 14 years of follow-up. These studies observed early motor delays following prenatal exposure to AP medication, which did not persist into later childhood. Five studies investigated risk for neurodevelopmental diagnoses and three studies explored school performance following prenatal AP exposure. No significant associations were found after adjustment for confounding. The quality of the evidence ranged from moderate to high.

**Conclusions:**

While the majority of studies did not identify differences between exposed and unexposed groups, some differences emerged early in infancy or when looking at neurodevelopmental disorders. However, our findings suggest that the observed neurodevelopmental differences are likely due to confounding by indication rather than exposure to antipsychotics themselves. More rigorous research is needed to clarify the neurodevelopmental effects of AP use during pregnancy.

**Disclosure of Interest:**

None Declared

